# Immunogenicity and predictive factors of hepatitis B vaccination with Fendrix^®^ in chronic kidney disease patients

**DOI:** 10.3389/fpubh.2025.1523733

**Published:** 2025-03-27

**Authors:** Cristina Hernán-García, Daniel Leonardo Sánchez-Carmona, Lucía Czestochowa Mateo-Otero, Virginia Fernández-Espinilla, Paula Andrea Rodriguez-Ducuara, José Javier Castrodeza-Sanz, Camino Prada-García

**Affiliations:** ^1^Preventive Medicine and Public Health Service, Hospital Clínico Universitario de Valladolid, Valladolid, Spain; ^2^Department of Preventive Medicine and Public Health, University of Valladolid, Valladolid, Spain; ^3^Department of Oncology, Hospital Universitario Río Hortega, Valladolid, Spain; ^4^Department of Dermatology, Complejo Asistencial Universitario de León, León, Spain

**Keywords:** chronic kidney disease, hepatitis B vaccine (HBV), vaccination, dialysis, observational

## Abstract

Hepatitis B virus (HBV) infection and chronic kidney disease (CKD) pose major global health challenges. CKD patients face a heightened risk of HBV infection, worsening their prognosis. This study evaluated the immune response to hepatitis B vaccination in CKD patients, the persistence of antibodies, and factors influencing vaccine efficacy. A retrospective study was conducted on 173 CKD patients (2014–2019) receiving routine vaccination at the Hospital Clínico Universitario de Valladolid, Spain. (ZIP Code:47003) Patients were immunized with Fendrix^®^ on a 0-1-2-6-month schedule, and verbal informed consent was obtained. A protective response was defined as Anti-HBs >10 IU/L, and a robust response as >100 IU/L. Overall, 90.8% achieved a protective response. Age was not a significant predictor (*p* = 0.137) between non-responders and protective or robust responders. 32.95% of patients died during follow-up. A robust response at the end of vaccination cycle was associated with higher antibody titers at 12 months (*p* = 0.002) but not at 24 (*p* = 0.550) or 36 months (*p* = 0.739). Kaplan–Meier analysis estimated median antibody duration as 26.5 months (Anti-HBs > 10 IU/L) and 25.4 months (Anti-HBs > 100 IU/L). A delay in vaccination compared to the recommended schedule was observed (one-sample Wilcoxon test, *p* < 0.001). Fendrix^®^ effectively induces protective immunity in CKD patients, but a robust early response does not ensure long-term persistence. The decline in antibody levels suggests the need for booster doses and periodic antibody monitoring to optimize long-term protection. Suboptimal vaccination adherence may reflect the inherent complexities of real-world clinical practice.

## Introduction

The World Health Organization (WHO) estimates that 254 million individuals suffered from chronic hepatitis B virus (HBV) infection in 2022, with 1.2 million new infections occurring annually (1).

In 2022, Hepatitis B (HB) caused about a million deaths, mainly attributed to cirrhosis or hepatocellular carcinoma (primary liver cancer) (1). In Europe, nearly all countries show annual incidence rates below 1 per 100,000, with the average in 2019 being 0.4 cases per 100,000 inhabitants (2).

In Spain, the annual incidence rate of HB is below 2 cases per 100,000 inhabitants. In 2023, 359 cases were reported, corresponding to an incidence rate of 0.50 cases per 100,000. Cases are predominantly detected in young adults, often among immigrant populations. Vertical transmission of the virus has been effectively eliminated through comprehensive public health strategies. The carrier prevalence in Spain is between 0.2 and 0.5%, categorizing it as a low-prevalence country. Hepatitis B is included in the Notifiable Diseases System of the National Epidemiological Surveillance Network (3).

HB and Chronic Kidney Disease (CKD) represent two major global health issues. Patients undergoing parenteral therapies for renal disease are at an elevated risk of HBV infection, as they are also immunocompromised (4, 5).

In Spain, vaccination against hepatitis B is included in the childhood vaccination schedule and is also recommended for certain high-risk groups. During infancy, it is administered at 2, 6, and 11 months of age (3). It is recommended for unvaccinated adults in high-risk groups or at-risk situations, such as healthcare professionals, individuals with HIV, and patients with renal or hepatic disease. In these cases, it is administered according to a four-dose schedule at 0, 1, 2, and 6-month intervals (6, 7).

High-risk groups such as pre-dialysis or dialysis CKD patients typically exhibit a lower -immune response to vaccination than immunocompetent individuals; therefore, higher antigen doses, the use of adjuvanted vaccines or more frequent booster doses may be required (8).

In Europe since 2005, Fendrix^®^, a recombinant DNA, adsorbed, and adjuvanted hepatitis B vaccine, has been recommended for patients with CKD. Each dose contains 20 mcg of HBsAg adjuvanted with the AS04C system, which aims to enhance humoral and cellular immunity. Additionally, it contains 50 mcg of 3-0-desacyl-4′-monophosphoryl lipid A (MPL) immersed in 500 mcg of aluminum phosphate and is produced using rDNA technology in *Saccharomyces cerevisiae* (9). This vaccine has demonstrated safety with no consistent evidence of any serious long-term sequelae (10–13).

In immunocompetent populations, seroprotection is defined as a titer of Anti-HBs greater than 10 IU/L (14). While a small proportion of healthy, immunocompetent adults fail to respond to the primary vaccination series, this proportion is significantly higher among stage V CKD patients and increases with disease progression. A second full vaccination series (3–4 doses) is recommended in these cases, achieving a protective antibody response in approximately 50% of additional patients (4, 5).

Hemodialysis patients who achieve adequate immune response following primary vaccination, may experience rapid antibody decline over time, returning to a susceptible state. Therefore, regular monitoring (yearly) for the presence of Anti-HBs is recommended, with administration of a booster dose if the titer falls below 10 IU/L (9).

The aim of this observational and retrospective study is to evaluate the vaccinal response, the duration of antibody persistence and associated factors in patients with chronic kidney disease (CKD) treated in the outpatient setting of the Preventive Medicine Service, to clarify the lack of evidence of the relevance of booster doses and periodic monitoring of antibodies.

## Methods

This retrospective observational study included 173 patients diagnosed with CKD, who were treated during routine clinical practice at the outpatient vaccination visit in the Preventive Medicine Service of the Hospital Clínico Universitario de Valladolid, Valladolid, Spain (Zip code: 47003). This study was conducted in the context of routine clinical practice, so in our setting, only verbal informed consent is required. These patients were referred for follow-up and vaccination regimen with Fendrix^®^ between 2014 and 2019. Data were collected through clinical records and vaccination history. The collected variables include demographic information, vaccination schedules, and serological results against HBV.

A convenience sample was taken, enrolling patients aged 18 years and older diagnosed with chronic kidney disease (CKD) at any stage. Patients with previous history and/or treatment of HBV infection, HIV, concomitant diseases causing immunodeficiency, use of immunosuppressants, pregnancy or history of allergic reactions to vaccination were excluded.

### Immunization

Previously unvaccinated patients received a full vaccination course with Fendrix^®^ (9). Each dose contained 20 micrograms of antigen, adjuvanted with AS04C, administered into the deltoid muscle, at the chosen starting date, then 1 month, 2 months, and 6 months thereafter.

The assessment of vaccination response involved analyzing Anti-HBs titers 4–8 weeks after the final vaccine dose. Further Anti-HBs titer measurements were done at 12, 24, and 36 months after completing the full vaccination course.

A “Protective response” was defined as Anti-HBs >10 IU/L, while a “robust response” was defined as Anti-HBs >100 IU/L (15). The immune response was analyzed independently in all patients using both cutoffs.

Patients categorized as non-responders (Anti-HBs <10 IU/L) after the initial vaccination series underwent a second cycle of immunization, with their response re-evaluated 1–3 months later. In routine clinical practice, a full second course of vaccination is not always given; rather, a single dose of the vaccine may be administered in exceptional circumstances.

### Statistical analysis

The anonymized database was provided by the Preventive Medicine and Public Health Service of the Clinical University Hospital of Valladolid.

In the descriptive analysis, means and standard deviations or medians and ranges were calculated for quantitative variables. For categorical variables, “*n*” counts and percentages were provided. In the bivariate analysis, the Chi-Square test was used to explore associations between categorical variables. The student’s *t*-test was used for ordinal categorical variables.

Binary logistic regression was performed to look for predictor variables of adequate immune response to vaccination. A statistical significance level of *p* < 0.05 was considered.

Descriptive and inferential statistical analysis was performed using the SPSS V23 statistical package (SPSS Software Inc., Chicago, Illinois, United States) And R Studio V2024.12.0 + 764 for graphics.

### Post-hoc analyses

We conducted three additional post-hoc analyses to further explore our findings. First, we calculated the median intervals between the first and second, third, and fourth doses and compared these to the recommended intervals (30, 60, and 180 days, respectively) using a one-sample Wilcoxon test. Next, we used a Mann–Whitney U test to determine whether patients who exhibited a robust response after the initial vaccination cycle maintained significantly higher antibody levels over time compared to those with only a protective response and adjusted the alpha level to 0.0175 using Bonferroni correction for multiple comparisons.

### Ethical considerations

This study adhered to the principles outlined in the Declaration of Helsinki (DoH) and complied with the prevailing data protection regulations. Verbal informed consent was obtained from all participants for study participation, blood collection, and vaccination, consistent with standard clinical procedures. Approval was granted by the Ethics Committee of the Valladolid East Health Area (CEIm) (code: PI: 19-1218).

## Results

**Figure 1:**
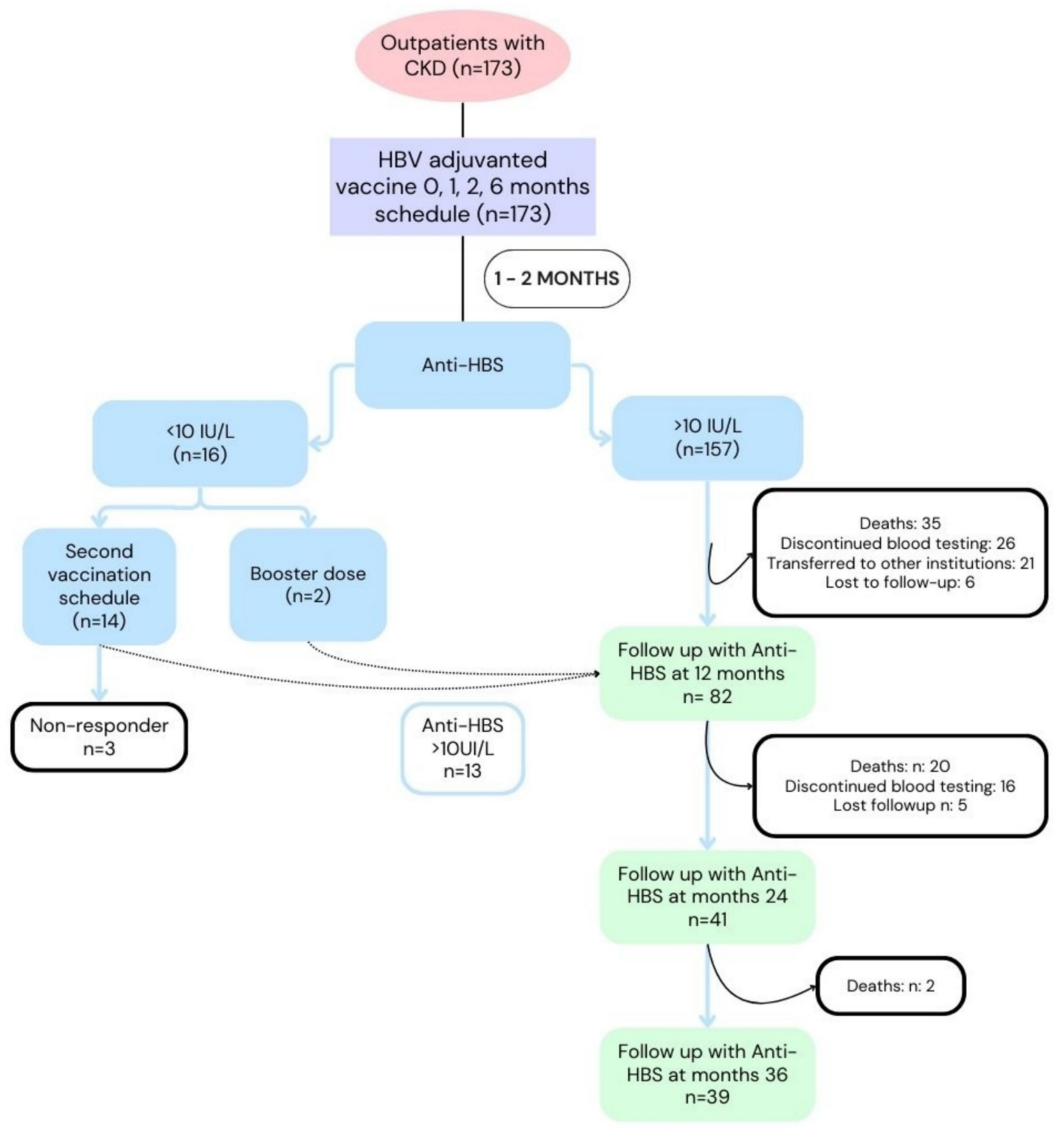
Patient adherence and outcomes following the Fendrix vaccination schedule in individuals with chronic kidney disease (CKD). This flowchart summarizes patient adherence, response rates, and outcomes across the study.

A total of 173 CKD patients received a first vaccination cycle with Fendrix^®^ (0, 1, 2, and 6 months schedule). Of these, 157 patients achieved an Anti-HBs response >10 IU/L, meeting the “protective response” cutoff. Of the 16 remaining patients with Anti-HBs <10 IU/L, 14 received a second vaccination course, while 2 received booster doses. Eventually, 13 of the 16 patients above seroconverted. The remaining 3 patients were ultimately classified as non-responders during the follow-up period, 57 patients died: 35 deaths occurred within 12 months, 20 within 24 months, and 2 within 36 months. Additional loss to follow-up included 42 patients who discontinued blood testing, 21 patients who transferred to other institutions, and 11 patients lost to follow-up for unspecified reasons. The final cohort available for analysis at 36 months consisted of 39 (Figure 1).

### Adherence and compliance with the regimen

The median intervals between doses were as follows: first to second 35 days (IQR = 10); first to third, 77 days (IQR = 29); first to fourth, 195 days (IQR = 40). We performed the Wilcoxon test for one sample, comparing the medians obtained for each interval with respect to the recommendation of 30 days between the first and second doses, 60 days between the first and third doses and 180 days between the first and fourth doses, obtaining *p* < 0.001 in all intervals.

### Immunogenicity

Among the three groups, the mean patient age was comparable, at 69.6 years for those with <10 IU/L, 70.1 years for 10–100 IU/L, and 65.7 years for >100 IU/L (*p* = 0.137). Most patients in all groups were older than 65 years, and 72% of these individuals achieved Anti-HBs levels >100 IU/L. Overall, 81.5% of the participants were male, with no significant difference in immune response by sex (males: 75.2%, females: 78.1%; *p* = 0.152).

Most patients were in pre-dialysis stage (84.4%), no differences were found in the Anti-HBs antibody response compared to the dialytic state (*p* = 0.224). The most common etiologies included diabetic nephropathy (22%) and unspecified causes (43.4%). A high proportion of patients in CKD stages 4 and 5 achieved Anti-HBs >100 IU/L (74.6 and 69%, respectively). CKD etiology and stage showed no statistically significant differences in Anti-HBs response (*p* = 0.666, *p* = 0.376 respectively) (Table 1).

**Table 1:** Baseline characteristics of patients and their Anti-HBs response categorized into three groups: <10 IU/L, 10–100 IU/L, and > 100 IU/L, after a full primary vaccination course.

Characteristics of patients		Total *n* = 173	Anti-HBs < 10 IU/L	Anti-HBs 10 to 100 IU/L	Anti-HBs > 100 IU/L	*p*-value
Average age in years (SD)		*N* (%)	69.6 years (±10.8)	70.1 years (±10.7)	65.7 years (±12.9)	*p* = 0.137
By age groups	<65 years	66 (38.1)	5 (7.6%)	7 (10.6%)	54 (81.8%)	*p* = 0.327
	>65 years	107 (61.8)	11 (10.3%)	19 (17.8%)	77 (72%)	
Gender, *n* (%)	Male	141 (81.5)	11 (7.8%)	24 (17%)	106 (75.2%)	*p* = 0.152
	Female	32 (18.5)	5 (15.6%)	2 (6.3%)	25 (78.1%)	
Predialysis/hemodialysis	Predialysis	146 (84.4)	14 (9.6%)	19 (13%)	113 (77.4%)	*p* = 0.224
	Hemodialysis	27 (15.6)	2 (7.4%)	7 (25.9%)	18 (66.7%)	
Etiology, *n* (%)	Unspecified	75 (43.4)	6 (8%)	14 (18.7%)	55 (73.3%)	*p* = 0.666
	Glomerulonephritis	9 (5.2)	1 (11.1%)	0	8 (88.9%)	
	Monorenal	6 (3.5)	1 (16%)	2 (33.3%)	3 (50%)	
	Lupus nephritis	2 (1.2)	1 (50%)	0	1 (50%)	
	Nephroangiosclerosis	16 (9.2)	1 (6.3%)	1 (6.3%)	14 (87.5%)	
	Diabetic nephropathy	38 (22)	2 (5.3%)	7 (18.4%)	29 (76.3%)	
	Tubulointerstitial nephropathy	8 (4.6)	2 (25%)	0	6 (75%)	
	Polycystic kidney disease	8 (4.6)	1 (12.5%)	0	7 (87.5%)	
	Others	11 (6.4)	1 (9.1%)	2 (18.2%)	8 (72.7%)	
CKD stage	CKD unspecified	42 (24.3)	1 (2.4%)	5 (11.9%)	36 (35.7%)	*p* = 0.376
	CKD 2	2 (1.2)	0	0 (0%)	2 (100%)	
	CKD 3	33 (19.1)	2 (6.1%)	8 (24.2%)	23 (69.7%)	
	CKD 4	67 (38.7)	9 (13.4%)	8 (24.2%)	50 (74.6%)	
	CKD 5	29 (16.8)	4 (13.8%)	5 (17.2%)	20 (69%)	

This table summarizes the demographic, clinical, and etiological characteristics of patients with chronic kidney disease (CKD) and their corresponding serological response to the Fendrix^®^ vaccination. The Anti-HBs response was stratified into three groups: <10 IU/L (non-responders), 10–100 IU/L (protective responders), and > 100 IU/L (robust responders).

In the multivariate analysis, no statistically significant differences were found between age over 65 years, sex and hemodialysis status and the variable “protective response” or “robust response (Annex I).

### Time course of antibody titers

**Figure 2:**
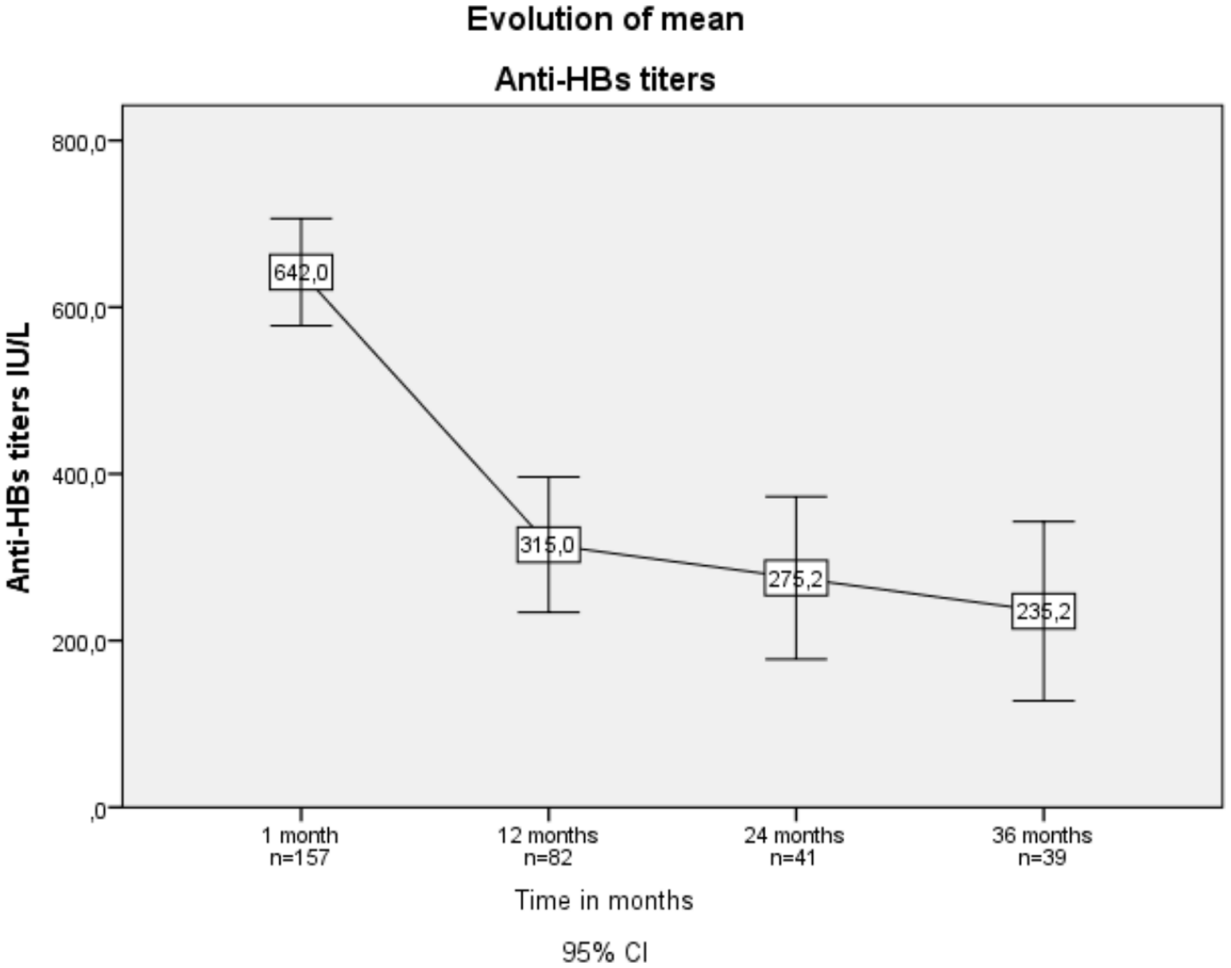
Evolution of mean Anti-HBs titers over time in vaccinated patients with chronic kidney disease.

The percentage of Anti-HBs >10 IU/L at month 1 was 90.75, 75.6% at month 12, 76.7% at month 24 and 71.9% at month 36. The mean Anti-HBs titers (IU/L) were 642 at 1 month (*n* = 157), 315 at 12 months (*n* = 82), 275.2 at 24 months (*n* = 41), and 235.2 at 36 months (*n* = 39) (Figure 2).

**Figure 3:**
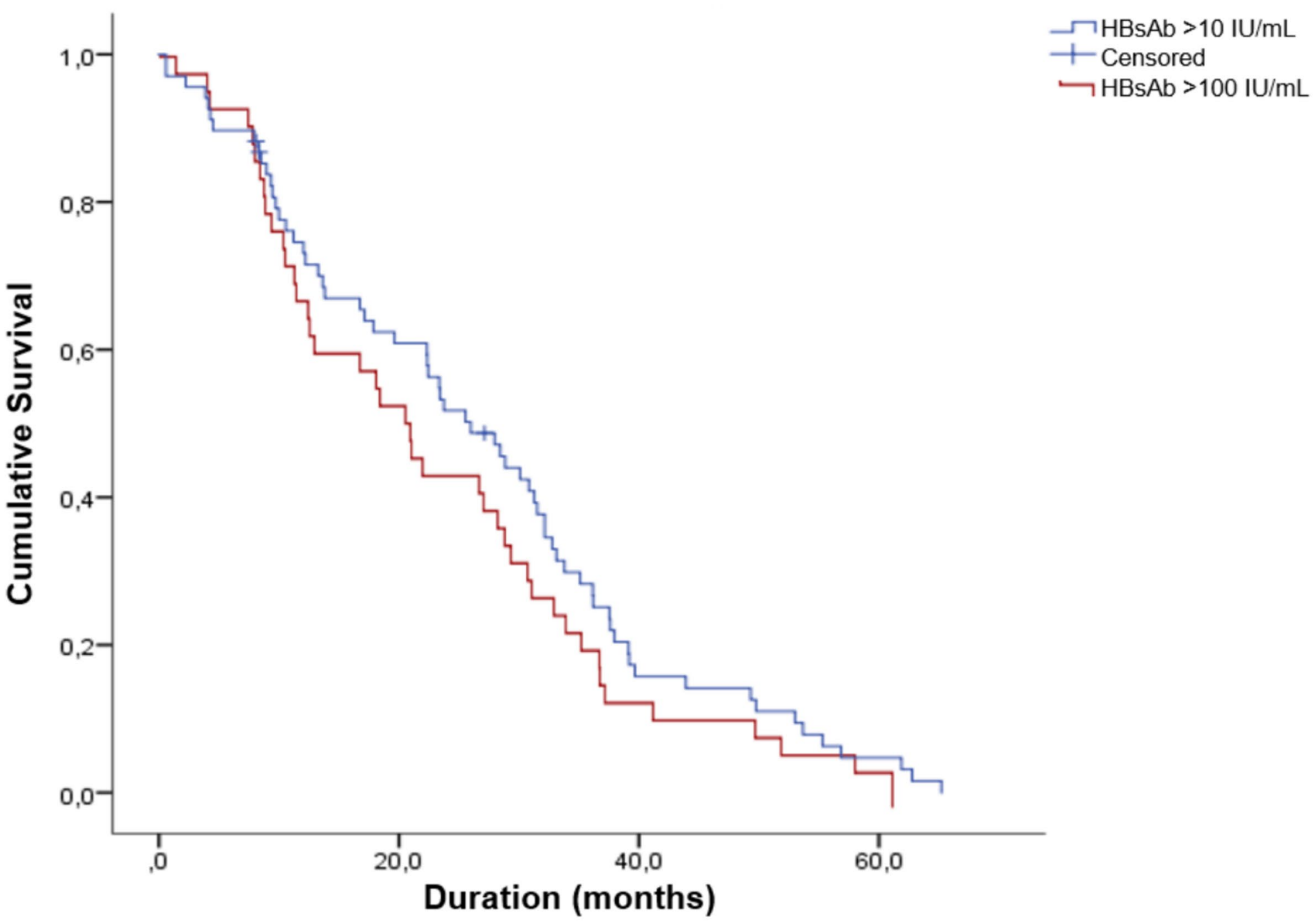
Kaplan–Meier survival analysis of Anti-HBs duration.

We estimated the duration of antibodies using a Kaplan–Meier analysis, finding that for the >10 UI/L cutoff, the median antibody duration was 26.5 months (95% CI = 22.4–30.5), and for the >100 UI/L cutoff, it was 25.4 months (95% CI = 20.2–30.5 months) (Figure 3).

**Figure 4:**
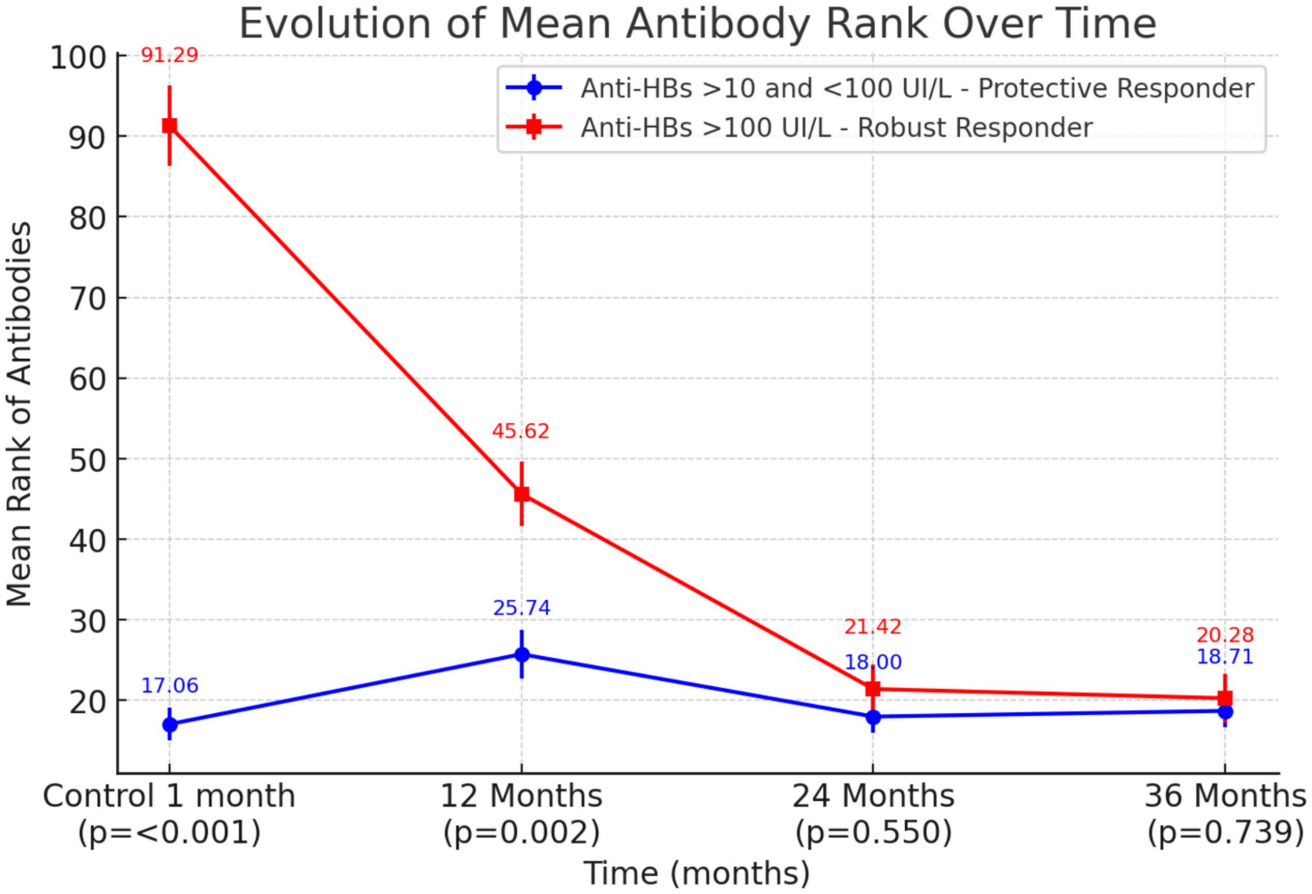
Evolution of mean antibody rank over time: mean rank (± standard error) of Anti-HBs titers for participants with protective (blue) vs. robust (red) responses at 1, 12, 24, and 36 months post-vaccination. *p*-values reflect comparisons made at an alpha level of 0.0175 after Bonferroni correction for multiple comparisons.

At 12 months, patients with a robust response exhibited a significantly longer duration of antibodies (*p* = 0.002). No statistically significant differences were observed at 24 months (*p* = 0.550) or 36 months (*p* = 0.739) (Figure 4; Annex II, III).

## Discussion

This study analyzed the response elicited by Hepatitis B virus (HBV) vaccination among individuals with chronic kidney disease in a real-world setting. The patients received the Fendrix^®^ vaccine, which contains the AS04C adjuvant, following the recommended schedule outlined in the product prescribing information (0-1-2-6 months).

A total of 173 patients with chronic kidney disease (CKD) 84.4% in the pre-dialysis stage and 15.6% in the dialysis stage were evaluated. In this cohort, stratifying patients into pre-dialysis and hemodialysis groups did not reveal significant differences in immune responses. These findings contrast with those reported by Light et al., who observed a stronger immune response in the pre-dialysis stage compared to the dialysis stage (16). However, those prior studies used non-adjuvanted vaccines, whereas adjuvanted vaccines have shown immune responses comparable to those of healthy individuals (12).

Scientific evidence shows that the adjuvanted AS04C vaccine tested in patients with CKD induces an earlier seroconversion at a higher rate compared to other non-adjuvanted vaccines. For instance, studies employing non-adjuvanted vaccines like Engerix B^®^ have documented lower seroconversion rates: Pereira et al. reported 56.9% (17), and Pin et al. observed 54.4% (18). In our study, we observed a seroconversion rate of 90.8%, consistent with the 91% rate reported by Tong et al. in their cohort using Fendrix^®^ (12). Our seroconversion rate surpasses the 84% rate reported by Fabrizi et al. in their cohort (19). Notably, another study by Fabrizzi et al. (13), conducted among pre-dialysis CKD patients, found a seroconversion rate of 95%. Additionally, the protective responses (Anti-HBs >100 IU/L) were more frequent in patients vaccinated with Fendrix^®^ (20, 21).

The cohort had a mean age of 69 years, with a notable predominance of male patients (81.5%). These findings align with established epidemiological trends of renal disease in our population, which show a higher prevalence among males and older age groups (22). In our study, age was not statistically significant in the vaccinal response (*p* = 0.137). We observed no significant sex-based differences between responders and non-responders, consistent with findings reported by Kim et al. (23).

In a review by Beran et al. (21), percentages of patients with Anti-HBs >10 IU/L at 12 months were reported at 86%, at 24 months at 80%, and 36 months at 80% post-vaccination. These figures slightly exceeded those observed in our sample, which were 75.6% at 12 months, 76.7% at 24 months, and 71.9% at 36 months.

The mean of Anti-HBs 1 month after completion of the regimen was 642.9 IU/L, 315 IU/L at 12 months, 275 IU/L at 24 months, and 235.2 IU/L at 36 months. These findings are consistent is with those found by Fabrizzi et al. (13) who found that Anti-HBs titers at 1 month after completing the regimen were 688.9 IU/L and 436.4 IU/L at 12 months.

Evidence indicates that higher antibody levels following primary vaccination are associated with the persistence of immunity over time (24). The statistical analysis suggests that while an initial robust response following primary vaccination is associated with significantly higher antibody after 12 months, these differences decline at 24 and 36 months. This shows that a robust early response does not necessarily ensure sustained antibody levels over time.

Patients were followed for a mean duration of 755 days (SD = 559). Using the Kaplan–Meier method, the mean duration time for a cut-off point of Anti-HBs >10 IU/L, was found to be 26.5 months (95% CI = 22.4–30.5), and for Anti-HBs >100 IU/L, it was 25.4 months (95% CI = 20.2–30.5). These findings suggest that antibody levels are maintained throughout the follow-up period, both for a protective response with antibody levels above 10 IU/L and for the maintenance of antibody levels above 100 IU/L in patients with kidney disease who received the 4-dose vaccination regimen with the adjuvanted Fendrix^®^ vaccine. However, observed gradual declines in titers underscore the need for periodic serological monitoring and potential booster vaccination to maintain immunity in this population.

A limitation of this study was the variable observation time, due to patient loss to follow-up from mortality (32.9%), as well as from other causes. Furthermore, although 173 patients were included, the sample size (particularly in specific subgroups such as those undergoing hemodialysis) may limit the ability to generalize the findings to the broader population with CKD. Future studies should aim to include larger samples to enhance statistical power and allow for greater differentiation between subgroups.

Furthermore, a delay was observed in the administration of successive vaccination doses according to the vaccination protocol, and this difference was statistically significant compared to the manufacturer-recommended dosing intervals. These findings, while suggesting an adherence issue, should be interpreted with caution due to clinical conditions inherent to CKD patients, such as comorbidities or disease exacerbations, as well as the logistical availability of vaccination centers.

Vaccination providers should adhere to the recommended intervals, although longer intervals generally do not reduce final antibody concentrations. The most advisable approach is to follow the dosing schedules outlined in the product prescribing information and based on clinical trial data, without the need to restart the series in case of interruption (25).

Finally, it is important to consider that younger generations will be vaccinated against HBV starting at birth or within 2 months, in accordance with the recommended immunization schedule (3). This early protection will precede the onset of renal disease in many cases. However, given the evident waning of protective antibodies over time, it is crucial to prioritize the development of updated, tailored vaccination programs for these patients. These programs should ensure an adequate immune response against hepatitis B throughout the patient’s lifetime and should also include or reinforce other vaccines targeting relevant pathogens.

## Conclusion

This study demonstrates that vaccination with Fendrix^®^ in patients with chronic kidney disease (CKD) effectively induces a protective immune response, with a high seroconversion rate. Our study did not identify any factors associated with a poorer vaccine response.

Robust early response does not necessarily ensure sustained antibody levels over time. Antibody levels remain relatively high up to 36 months post-vaccination, though they progressively decline. This observation highlights the importance of periodic serological monitoring and booster doses for those with non-protective Anti-HBs levels to maintain long-term protection. Furthermore, systematic vaccination from birth could improve initial protection against hepatitis B virus (HBV) before the onset of kidney disease, underscoring the importance of keeping vaccination programs up to date for these patients.

These findings highlight the need to redefine vaccination strategies, particularly regarding booster dose recommendations. The observed decline in antibody levels over time suggests that additional boosters may be required to maintain long-term immunity. Further research is necessary to explore the immunological mechanisms influencing antibody persistence and to determine optimal vaccination schedules for prolonged protection.

### Future perspectives

A follow-up of this cohort at 60 months is currently being conducted to further analyze the long-term persistence of immunity.

Future studies could explore how vaccination with Fendrix^®^ impacts not only immunogenicity but also the reduction of liver complications, overall morbidity, or even mortality in CKD patients. Additionally, a cost-effectiveness evaluation should be considered, comparing Fendrix^®^ with other vaccines, considering the need for booster doses and the broader long-term health outcomes, including the reduced risk of hepatitis B in this vulnerable population.

## Data Availability

The raw data supporting the conclusions of this article will be made available by the authors, without undue reservation.
