# Supplementary material for: Immunogenicity and predictive factors of hepatitis B vaccination with Fendrix® in chronic kidney disease patients
Source: Front Public Health. 2025 Mar 27;13:1523733. doi: 10.3389/fpubh.2025.1523733 (PMC11983650; doi:10.3389/fpubh.2025.1523733)
Supplement: Supplementary file 1 [file Data_Sheet_1.docx]

Annex Ia. Multivariant analysis for Anti-HBs> 10 UI/L.

| Variables | Odds ratio | C.I 95% | P value |
| --- | --- | --- | --- |
| Sex | 2,182 | 0,70-6,80 | 0,179 |
| Age > 65 years | 0,836 | 0,429-4,25 | 0,608 |
| Pre-dialysis | 1,350 | 0,169-4,137 | 0,826 |

Annex Ib. Multivariant analysis for Anti-HBs> 100 UI/L

|  | Odds ratio | C.I 95% | P value |
| --- | --- | --- | --- |
| Sex | 1,134 | 0,447-2,87 | 0,791 |
| Age > 65 years | 0,507 | 0,229-1,12 | 0,094 |
| Pre-dialysis | 0,460 | 0,180-1,11 | 0,105 |

**Annex II: Kolmogorov-Smirnov Test**

| **Time Point** | **Kolmogorov-Smirnov Z** | **p-value** | **Interpretation** |
| --- | --- | --- | --- |
| **1 Months** | 4.479 | **0.000** | **Not normal** (p<0.05) |
| **12 Months** | 1.771 | **0.004** | **Not normal** (p<0.05) |
| **24 Months** | 0.664 | **0.771** | **Normal** (p>0.05) |
| **36 Months** | 0.481 | **0.975** | **Normal** (p>0.05) |

**Annex III:** **Mann-Whitney U Test.**

| **Time Point** | **Group 0 (non-robust)** | **Mean Rank** | **Group 1 (Robust)** | **Mean Rank** | **p-value^a^** |
| --- | --- | --- | --- | --- | --- |
| **1 Month (control)** | 26 | **17.06** | 131 | **91.29** | **<0.001** |
| **12 Months** | 17 | **25.74** | 65 | **45.62** | **0.002** |
| **24 Months** | 5 | **18.00** | 36 | **21.42** | 0.550 |
| **36 Months** | 7 | **18.71** | 32 | **20.28** | 0.739 |

a. The alpha level was set to 0.0175 after Bonferroni correction for multiple comparisons.
